# Screening of Stepping Forces in an Arthritic Rat Model Using a Novel Analgesic Meter and Data Acquisition System

**DOI:** 10.3390/s120709603

**Published:** 2012-07-16

**Authors:** Mun Fei Yam, Mariam Ahmad, Lip Yee Por, Lee Fung Ang, Rusliza Basir, Mohd. Zaini Asmawi

**Affiliations:** 1 School of Pharmaceutical Sciences, Universiti Sains Malaysia, Penang 11800, Malaysia; E-Mails: mariam@usm.my (M.A.); ang.leef@gmail.com (L.F.A.); amzaini@usm.my (M.Z.A.); 2 Faculty of Computer Science and Information Technology, University of Malaya, Kuala Lumpur 50603, Malaysia; E-Mail: porlip@um.edu.my; 3 Unit of Pharmacology, Department of Human Anatomy, Faculty of Medicine and Health Sciences, Universiti Putra Malaysia, Selangor 43400, Malaysia; E-Mail: rusliza@medic.upm.edu.my

**Keywords:** arthritis, analgesic meter, nociception, stepping force, load cells

## Abstract

The stepping forces of normal and Freund Complete Adjuvant (FCA)-induced arthritic rats were studied *in vivo* using a proposed novel analgesic meter. An infrared charge-coupled device (CCD) camera and a data acquisition system were incorporated into the analgesic meter to determine and measure the weight of loads on the right hind paw before and after induction of arthritis by FCA injection into the paw cavity. FCA injection resulted in a significant reduction in the stepping force of the affected hind paw. The stepping force decreased to the minimum level on day 4 after the injection and then gradually increased up to day 25. Oral administration of prednisolone significantly increased the stepping forces of FCA-induced arthritic rats on days 14 and 21. These results suggest that the novel device is an effective tool for measuring the arthritic pain in *in vivo* studies even though walking is a dynamic condition.

## 1. Introduction

Arthritis is a degenerative and debilitating disease that is associated with chronic pain in the joints, and can impair the ability to work and also lead to severe psychological and social problems. Osteoarthritis is suffered by 15% of the World's population, whereas rheumatoid arthritis, a chronic inflammatory illness, affects about 1% of the World's population [1–3]. Behavioural tests are used in animal models to provide valuable information about the pain mechanism and potential pharmacological therapies. However, in some cases, these tests, which include the paw withdrawal threshold and latency [4–6] and the walking on a rotating cylinder methods in arthritic rats [7], are not suitable because stressful manipulations are involved. Likewise, the withdrawal response to radiant heat method is not suitable as heat pain differs from arthritic pain [8]. Therefore, a weight distribution model that uses sensors to capture the weight load of an animal was proposed [9]. The state of arthritic pain can be determined by measuring the weight load while the rat is walking freely because weight bearing is reduced in arthritic animals when walking [10]. However, in this protocol the rat is not restrained nor forced to maintain its static position [10]. Therefore, it is difficult to identify the correct paw of the rat when using the device, especially given the lack of a camera to capture real-time movements, which might affect the peak signal captured. Thus for example, the device might capture the signal produced by the front paw or the hind paw or even both paws of the rat. Furthermore, the stepping force and movement of the rodent cannot be synchronized due to the inflexible nature of the proprietary software used. Hence, a novel apparatus was developed to measure the stepping force of rodents. This apparatus was fabricated with a built-in infrared CCD camera integrated within the analgesic meter [7]. This camera captures the locomotion of the rats and synchronizes the stepping force concurrently. Using this feature, the steps produced by the rat can be correctly identified and the stepping force caused by the front paw can be differentiated from that of the hind paw [11].

In this study, we aimed to establish a simple and reproducible method of paw pain assessment using the stepping force analgesic meter in the FCA-induced arthritis model over a 25-day period with a view to using this method to rapidly evaluate therapeutic drugs in future studies. This model resembles asymmetric monoarthritis in which only one knee joint is affected. We hypothesized that there would be a change in stepping force in these animals in the ipsilateral affected limb leading to asymmetry in the stepping force.

## 2. Methodology

### 2.1. Experimental Animal

Male Sprague Dawley rats weighing 180−200 g were used throughout the study. The animals were obtained from the Animal House Facility, School of Pharmaceutical Sciences, Universiti Sains Malaysia (USM). They were maintained at a room temperature of 28−30 °C and allowed access to food (normal laboratory chow) and tap water *ad libitum*. The animals were acclimatized to laboratory conditions for 7 days before the commencement of experiments. All procedures in this study were performed in accordance with the Animal Ethics Committee Guidelines of USM.

### 2.2. Fabrication of the Analgesic Meter

There are four main components in the analgesic meter: (i) an apparatus, (ii) an amplifier station, (iii) a video camera box, and (iv) a computer. The apparatus contains a starting box, a tunnel and an arrival box. There are eight transparent Perspex plates (width × length: 5 cm × 7 cm) along the tunnel, and each plate is attached to a load cell (strain gauge type, working range 0–600 g, DA cell, Korea). An analog amplifier (AM 100) (DA cell, Korea) is used to amplify the signal captured by each load cell. An LCPI 9112 analog–digital converter (Adlink Tech. Inc., Taiwan) conducts the amplified signal to a personal computer. A Bakelite box hosts a Huper H4MR type IR CCD camera (Huper, USA). A Picolo PCI card (Euresys, USA) is used to transfer the captured image from the CCD camera to the personal computer [10]. The computer is used to gather and process the data acquired by the analgesic meter. The data acquisition software was programmed using Visual Basic 6 [10]. The data acquisition software allows researchers to customise their *in vivo* studies based on individual requirements. The signal produced by the stepping force of a rat can be saved in ASCII text file format with the extension “.rdf”. Similarly, the image captured and the video recorded can be saved in AVI format. These saved files can then be used for further analysis for research in arthritis [10].

### 2.3. In Vivo Study

**Figure 1.**
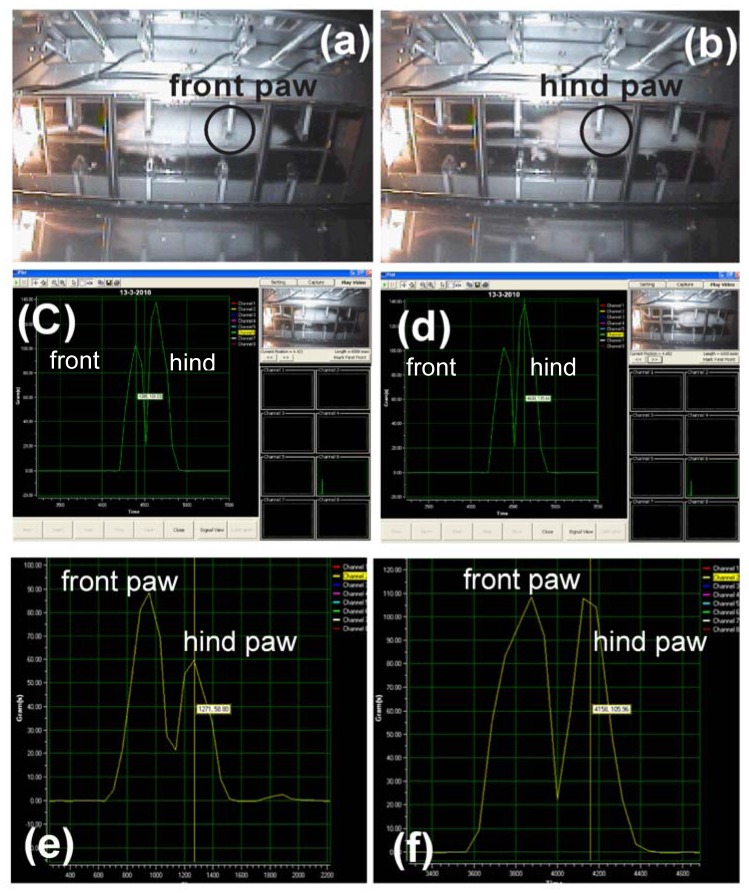
Walking steps of rat were captured by CCD camera, (**a**) and (**b**) showed the image of front and hind paws. Typical stepping force *versus* time curves constructed and for normal hind and front paws before the induction of arthritis (**c**,**d**), after the induction of arthritis by FCA injection (**e**) and in the FCA-induced arthritic rats treated with prednisolone (**f**). In normal rats, the stepping force produced by the hind paw was always higher than that of the front paw (c,d). After the induction of arthritis, the stepping force produced by the affected hind paw decreased (e). There was an improvement in the stepping force in rats treated with prednisolone (f).

An *in vivo* study was carried out using normal healthy male Sprague Dawley (SD) rats (N = 6). A rat was randomly selected, placed in the starting box and allowed to walk freely along the sensor tunnel until it reached the arrival box. The test was repeated at least three times a day for a total of six days. For the arthritis study, another 24 male rats were used as test animals. Arthritis was induced by intradermal injection of 0.1 mL FCA (Sigma, USA) into the right hind paw of each rat. On the 4th day after the injection of FCA, the rats were randomly divided into groups of six and treated as follows: group A, the control group, was treated with saline (10 mL/kg per day, p.o.); group B was treated with prednisolone at 10 mg/kg per day, p.o.; group C was treated with prednisolone at 5 mg/kg per day, p.o.; group D was treated with prednisolone at 2.5 mg/kg per day, p.o. The rats were placed in the starting box and allowed to walk voluntarily along the sensor tunnel until they reached the arrival box. Inflammation was assessed by measuring the right ankle thickness with a micrometer one day prior to the induction of arthritis, and 4, 7, 14, 21 and 25 days after induction. The stepping forces of the rats were also measured using the proposed analgesic meter the day before the induction of arthritis, and 4, 7, 14, 21 and 25 days after induction. The stepping force signals produced were recorded and interpreted (Figure 1).

### 2.4. Statistical Analysis

One-way analysis of variance (ANOVA) was performed using the Statistical Package for Social Sciences (SPSS) (Chicago, IL, USA). Significant differences between the means of groups were determined using the least significant difference multiple comparison test, and *P* < 0.05 was accepted as significant.

## 3. Results

### 3.1. Precision and Accuracy of the Analgesic Meter

The intra- and inter-day precision and accuracy of the measurements made by each channel of the load cell are shown in Table 1. The %RSD values for intra- and inter-day measurements were 0.08–0.68 and 0.52–0.88, respectively. The accuracy for intra- and inter-day measurements was 99.68–100.69 and 99.42–100.36, respectively.

### 3.2. In Vivo Study

#### 3.2.1. Normal Rat

**Figure 2.**
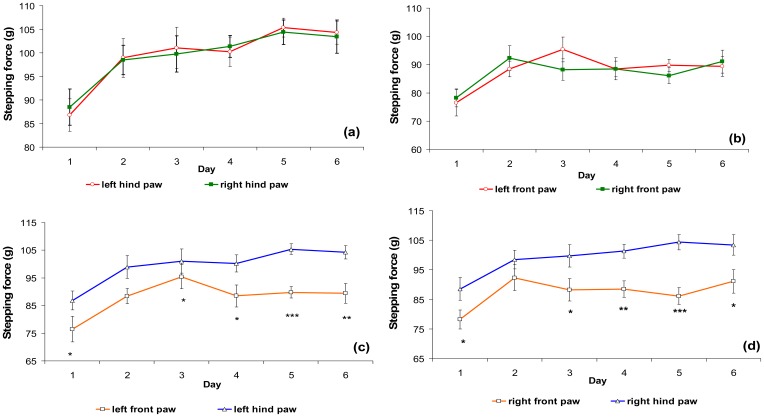
Comparison of stepping forces. (**a**) Left and right hind paws, (**b**) left and right front paws, (**c**) left front and hind paws, (**d**) right front and hind paws. (N = 6) * *P* < 0.05, ** *P* < 0.01, *** *P* < 0.001 between the groups.

The animals were tested over a period of six days to evaluate the effect of repeated measurement between days. On each test day, the stepping force in consecutive tests on both the left and right sides and hind and front paws of the rats was recorded (Figure 2).

**Figure 3.**
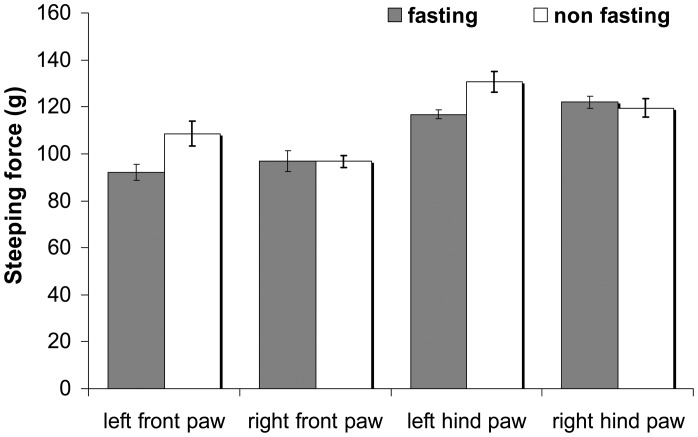
Comparison of stepping forces of the left and right, front and hind paws of fasting and non-fasting rats.

There was no significant difference in the stepping force between the left and right hind paw, or the left and right front paw in fasting and non-fasting conditions. However, there was a significant difference between the left hind and front paw, and right hind and front paw (Figure 3).

#### 3.2.2. FCA-Induced Arthritic Rats

**Figure 4.**
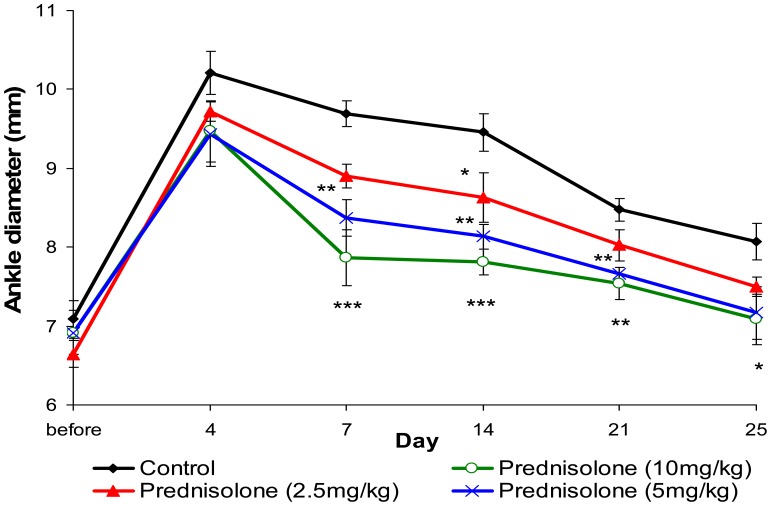
Anti-inflammatory effect of prednisolone on FCA-induced arthritic rats. (N = 6) * *P* < 0.05, ** *P* < 0.01, *** *P* < 0.001 compared to the control group.

Unilateral injection of FCA into the subcutaneous cavity of the rats' heel produced pronounced edema in the ipsilateral (right hind paw) ankle as shown by the substantial increase in diameter of this ankle compared to basal values taken before the induction of arthritis on day 0 (Figure 4).

Swelling was observed on day 1 and reached a maximum on day 4 after induction of arthritis; it then gradually diminished but the ankle had not returned to its original diameter by day 25. Oral treatment with prednisolone (10 and 5 mg/kg per day) for 21 days succeeded in reducing the swelling from day 7 onwards.

**Figure 5.**
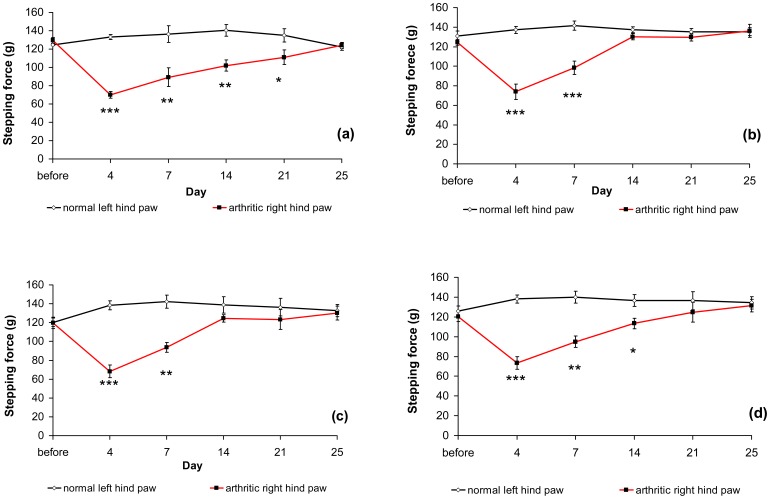
Stepping forces of right hind paws of FCA-induced arthritic rats. (**a**) Untreated, (**b**) orally treated with prednisolone at 10 mg/kg per day, (**c**) orally treated with prednisolone at 5 mg/kg per day, (**d**) orally treated daily with prednisolone at 2.5 mg/kg per day. (N = 6) * *P* < 0.05, ** *P* < 0.01, *** *P* < 0.001 compared to the stepping force of the normal left hind paw in the same group.

Subcutaneous injection of FCA into the right hind paw resulted in a significant reduction in the stepping force of the injected leg on day 4 (Figure 5). The time course of the change in stepping force is illustrated in Figure 5. On day 4 after the injection of FCA, the stepping force of the affected leg was lower than that of the non-affected leg. It subsequently showed a gradual increase, returning to the pre-injection level by day 25 (Figure 5(a)). Prednisolone at doses of 5 and 10 mg/kg per day improved the stepping forces, returning them to their original level by day 14. However, prednisolone at 2.5 mg/kg per day did not lead to a significant improvement in stepping forces until day 21.

## 4. Discussion

The validation of each channel showed convincing results whereby intra-day and inter-day precision were less than 1% and accuracy was 99.42–100.69%. The low %RSD (<2%) reflected the high precision of the analgesic meter and all accuracy tests were within 99–101%, indicating that the analgesic meter is both sensitive and highly accurate.

An *in vivo* study on normal rats was conducted prior to investigating the effectiveness of the analgesic meter for arthritic rats. The normal male rats were allowed to walk repeatedly along the sensor tunnel over a period of six days. The stepping forces of the left and right paws (hind and front) were recorded. The results (Figure 2) clearly showed that there were no significant differences between the stepping forces of the left and right hind paws, or of the left and right front paws. This means that normal rats distribute their stepping forces equally between their left and right paws. An *in vivo* study was then conducted in fasting and non-fasting normal male rats. These were allowed to walk along the sensor tunnel and the stepping forces were recorded. The results showed that there were no significant differences between non-fasting and fasting rats (Figure 3).

FCA-induced arthritis is a well-established model that has been used in numerous studies to investigate the pathogenesis of rheumatoid arthritis and to identify potential therapeutic targets. FCA arthritis is very similar to human rheumatoid arthritis both in terms of pathological and serological changes [11], including the involvement of inflammatory mediators in the arthritis etiology. In this study, we found that all injected ankles became swollen and appeared red on the second day after FCA injection. However no secondary reaction was observed in the left foot and forelimbs. After 11 days (14 days after FCA injection) of treatment with prednisolone at doses of 5 and 10 mg/kg per day, the swelling in the right ankle was significantly lower than that in the control group. According to Gao *et al.* injection of FCA causes a histopathological change in the ankle joint, with anomalous hyperplasia of the synovial membranes, which is one of the principal factors contributing to joint damage in rheumatoid arthritis [12].

The subcutaneous injection of FCA into the right hind paw resulted in a significant reduction in the stepping force of the injected leg. The time course of the changes in stepping force is illustrated in Figure 5. On day 4 after the injection of FCA, the stepping force of the affected leg was lower than that of a normal leg. Thereafter, it gradually increased, returning to the pre-injection level by day 25 (Figure 5). The ability of the analgesic meter to detect FCA-evoked hypersensitivity shows that it is an excellent experimental model in which to test the analgesic properties of novel compounds during the steady inflammatory state between days 14 and 21 post-FCA (Figure 5). Prednisolone, a standard drug that acts as a positive control, was administered over a 21-day period to determine whether the drug would lead to the resolution of FCA-induced hypersensitivity, reflecting the hyperalgesia that we would expect to see in humans. The analgesic meter detected prednisolone analgesia, over the 21 days of dosing, which decreased after dosing ceased.

A decrease in stepping force is one of the most commonly observed functional disabilities in arthritic animals and humans. The weight load or stepping force of an arthritic leg shows the most prominent change among various parameters including swing time, length of stride and velocity of locomotion in gait analysis [13]. Moreover, the weight load or stepping force of an arthritic limb in the standing position can be measured with reproducibility and is a good index for the severity of arthritic pain in the rat [14]. The current device was fabricated so that the rat was made to walk voluntarily. It was neither restrained in a cage nor forced to maintain a standing position. Another advantage of this device is that it reflects pain levels more realistically compared to previous devices, as it measures stepping force and records every movement while the rat is walking.

## 5. Conclusions

The fabricated device includes a new feature, that is, a built-in infrared CCD camera. The camera is able to capture the locomotion of the rats and synchronise the stepping force concurrently so that each step can be identified and interpreted using the data acquisition system. The fabricated analgesic meter showed good repeatability and reliability. In conclusion, we have constructed a device that allows easy measurement of the stepping force of a voluntarily walking rat. Using this device, we showed that the weight load is reduced in legs in which arthritis was induced by FCA injection and that this reduction could be reversed by the oral administration of prednisolone. This indicates that the device is an effective tool for the assessment of arthritic pain within a more realistic setting, and may be useful for discerning therapeutic approaches considered worthy of further investigation using other arthritic animal models.

## Figures and Tables

**Table 1.** Precision and accuracy of each channel of the analgesic meter.

**Channel**	**Intra-day**		**Inter-day**	

	**%RSD**	**Accuracy**	**%RSD**	**Accuracy**
1	0.16	99.90	0.66	100.10
2	0.12	99.99	0.52	100.01
3	0.58	100.41	0.73	100.16
4	0.08	99.68	0.56	99.66
5	0.58	99.71	0.70	99.87
6	0.34	100.69	0.71	100.36
7	0.68	99.87	0.88	99.91
8	0.35	100.05	0.69	99.42
